# Supplementary material for: A comparison of food sources of nudibranch mollusks at different depths off the Kuril Islands using fatty acid trophic markers
Source: PeerJ. 2021 Nov 24;9:e12336. doi: 10.7717/peerj.12336 (PMC8627124; doi:10.7717/peerj.12336)
Supplement: Supplemental Information 2 — Values show the contribution of individual FAs to the percent similarity or dissimilarity of the group. Total similarity or dissimilarity between groups is given in bold. TT, Tritonia tetraquetra; CP, Colga pacifica; CV, Coryphella verrucosa; DS, Dendronotus sp.; AP, Aeolidia papillosa. [file peerj-09-12336-s002.docx]

**Supplement materials**

A comparison of food sources of nudibranch mollusks at different depths off the Kuril Islands using fatty acid trophic markers

Anatolii A. Komisarenko, Vladimir V. Mordukhovich, Irina A. Ekimova, Andrey B. Imbs

**TABLE S2** **Results of SIMPER analysis of the FA composition data for the five nudibranch species.** Values show the contribution of individual FAs to the percent similarity or dissimilarity of the group. Total similarity or dissimilarity between groups is given in bold. TT, *Tritonia tetraquetra*; CP, *Colga pacifica*; CV, *Coryphella verrucosa*; DS, *Dendronotus* sp.; AP, *Aeolidia papillosa*.

|  | CV |  | DS |  | CP |  | TS |  | AP |  | TT |  |
| --- | --- | --- | --- | --- | --- | --- | --- | --- | --- | --- | --- | --- |
| CV | **Simil.**  20:5n-3  16:0  22:6n-3 | **62.8**  28.0  15.0  8.6 | **Dissimil.**  20:5n-3  20:1n-9  18:0  20:4n-6 | **35.6**  15.0  8.5  7.7  7.1 | **Dissimil.**  20:5n-3  22:5n-6  Δ7,13-22:2 | **50.9**  18.2  11.4  10.1 | **Dissimil.**  24:6n-3  20:5n-3  Δ7,13-22:2  20:4n-6  20:1n-9 | **49.9**  13.7  11.7  9.5  8.9  7.1 | **Dissimil.**  20:5n-3  20:1n-9  22:5n-3 | **38.5**  15.2  8.8  7.9 | **Dissimil.**  20:4n-6  20:5n-3  24:6n-3  24:5n-6 | **54.8**  14.6  13.7  8.6  7.2 |
| DS |  |  | **Simil.**  20:5n-3  16:0  22:6n-3  18:0  20:4n-6 | **83.8**  21.4  13.7  12.6  8.9  7.9 | **Dissimil.**  20:5n-3  22:5n-6  Δ7,13-22:2 | **40.5**  13.5  13.4  9.4 | **Dissimil.**  24:6n-3  22:6n-3  Δ7,13-22:2 | **35.0**  20.4  14.8  9.8 | **Dissimil.**  22:5n-3  16:0  20:4n-6 | **29.8**  10.8  8.4  7.5 | **Dissimil.**  20:4n-6  22:6n-3  24:6n-3  24:5n-6  20:5n-3 | **41.2**  13.3  12.6  12.2  9.6  9.1 |
| CP |  |  |  |  | **Simil.**  22:6n-3  16:0  Δ7,13-22:2  20:5n-3 | **71.3**  13.4  11.9  10.8  7.9 | **Dissimil.**  24:6n-3  22:5n-6  22:6n-3  20:5n-3  20:4n-6 | 47.1  14.8  12.3  12.1  8.8  8.3 | **Dissimil.**  22:5n-6  Δ7,13-22:2  20:5n-3  22:5n-3 | **37.1**  13.1  11.0  10.9  9.6 | **Dissimil.**  20:4n-6  22:6n-3  22:5n-6  24:6n-3  24:5n-6 | **50.6**  14.8  11.2  10.9  9.6  7.3 |
| TS |  |  |  |  |  |  | **Simil.**  20:5n-3  24:6n-3  16:0  20:4n-6  Δ7,13-22:2 | **88.8**  16.5  15.6  13.8  12.4  10.0 | **Dissimil.**  24:6n-3  20:4n-6  22:6n-3  22:5n-3  Δ7,13-22:2 | **44.3**  16.0  9.3  8.7  8.4  8.4 | **Dissimil.**  20:4n-6  24:5n-6  20:5n-3  24:6n-3  16:0 | **19.3**  18.6  13.3  12.3  10.9  8.5 |
| AP |  |  |  |  |  |  |  |  | **Simil.**  20:5n-3  22:6n-3  22:5n-3  16:0 | **88.0**  15.4  9.4  8.1  8.0 | **Dissimil.**  20:4n-6  24:6n-3  16:0  22:5n-3  24:5n-6 | **48.9**  15.8  10.2  9.2  8.1  7.9 |
| TT |  |  |  |  |  |  |  |  |  |  | **Simil.**  20:4n-6  16:0  20:5n-3  24:6n-3  24:5n-6 | **90.6**  20.4  17.5  11.6  9.9  7.8 |
