# Supplementary figures and images for: A comparison of food sources of nudibranch mollusks at different depths off the Kuril Islands using fatty acid trophic markers
Source: PeerJ. 2021 Nov 24;9:e12336. doi: 10.7717/peerj.12336 (PMC8627124; doi:10.7717/peerj.12336)

Non-metric MDS

Resemblance: S17 Bray-Curtis similarity

2D Stress: 0,06

Species

- ▲ CV
- ▼ AP
- CP
- ◆ TS
- TS-
- + DS

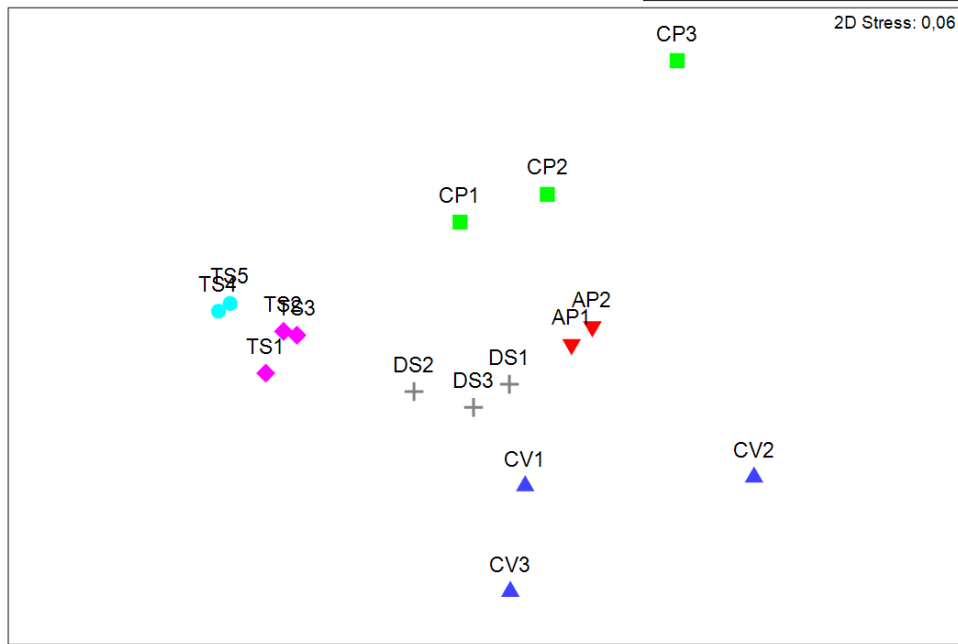

Supplement: Supplemental Information 3 — AP, Aeolidia papillosa; CV, C. verrucosa; CP, C. pacifica; DS, Dendronotus sp.; TS1–TS3, T. tetraquetra specimens from a depth of 450–516 m; TS4 and TS5, T. tetraquetra specimens from a depth of 210–247 m. [file peerj-09-12336-s003.pdf]

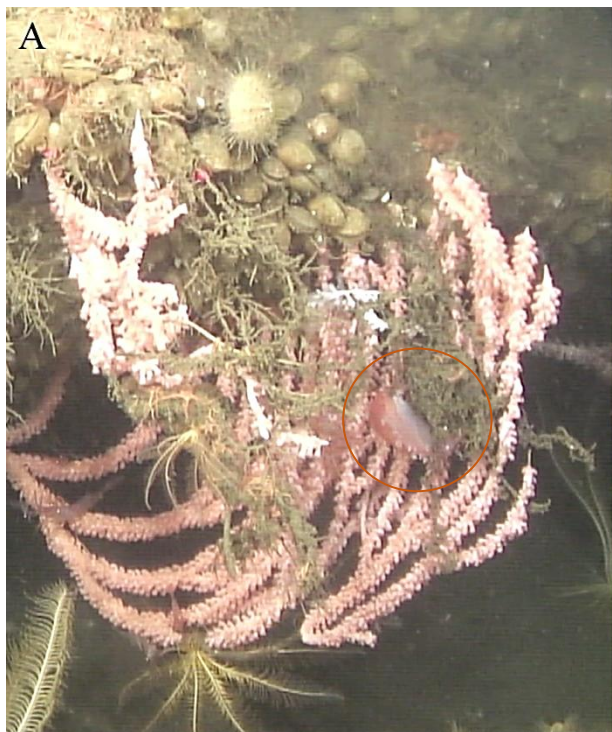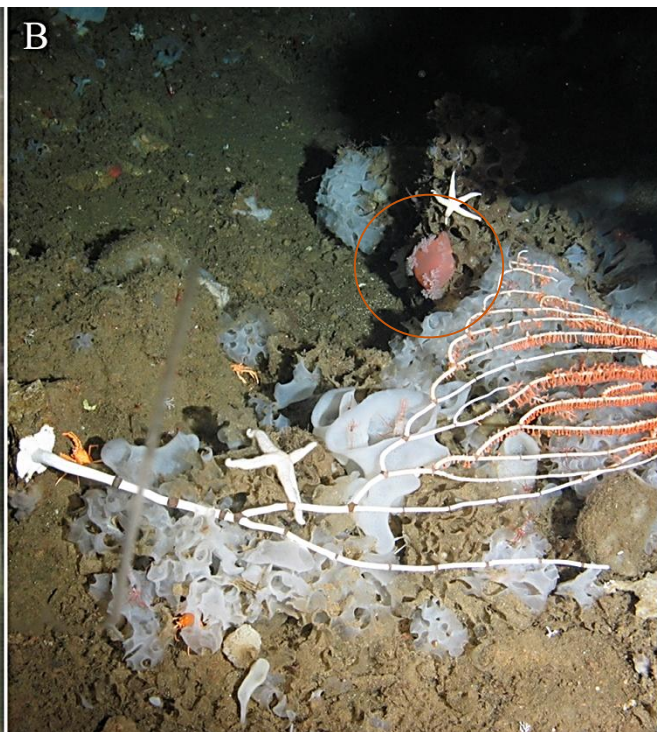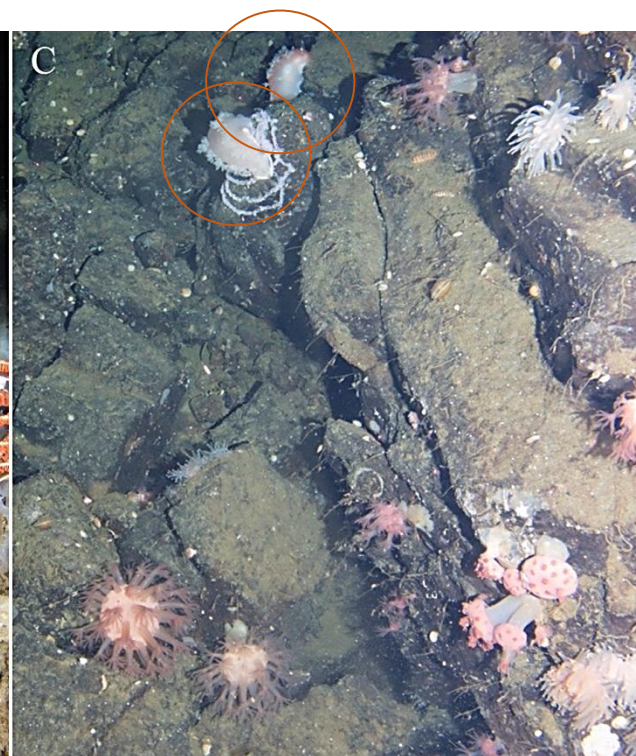

Supplement: Supplemental Information 4 — A, the mollusk on the Primnoa corals, the Sea of Japan, depth 676 m; B, the mollusk on the Primnoa corals, the Sea of Japan, depth 676 m; C, the mollusk near Heteropolypus rylovi and Corallimorphus pilatus, the Bering Sea, depth 381 m. The mollusks are pointed by cycles. [file peerj-09-12336-s004.pdf]
